# Green state entrepreneurialism: Building the park city in Chengdu, China

**DOI:** 10.1177/27541223241253231

**Published:** 2024-06-03

**Authors:** Fangzhu Zhang, Fulong Wu

**Affiliations:** Bartlett School of Planning, University College London, UK; Bartlett School of Planning, University College London, UK

**Keywords:** Environmental governance, green infrastructure, ecological civilisation, state entrepreneurialism, Chengdu

## Abstract

This paper uses the perspective of state entrepreneurialism to explore China’s environmental governance. The perspective illustrates how the Chinese state maintains its centrality, combining environmentalism and developmentalism while deploying flexible market development tools. This paper examines the Chengdu park-city model, an exemplar President Xi Jinping endorsed and widely emulated in China. The model combines the development of industrial and ecological spaces. It aims to deliver the central government’s vision for ecological civilisation and the local government’s economic development strategy. The development tools include land consolidation, financial mobilisation and an economic strategy that attempts to introduce ‘urban scenes’ into ecological spaces. This ecologically oriented development approach is more state-centred, contrasting with the neoliberal green growth machine.

## Introduction

China has seen experiments with eco-cities and low-carbon development (Chang et al., 2016; Chung and Xu, 2021; Liu and Lo, 2023; Xie et al., 2019) after tightening environmental regulations (Geall and Ely, 2018; Kostka and Nahm, 2017; Kostka and Zhang, 2018). Some earlier eco-cities pursued land-driven development and became ‘greenwashing’ as their ecological value is rather dubious (Caprotti et al., 2015; Chien, 2013). Faced with environmental crises, some cities attempt to fix the ecology by investing in green infrastructures and promoting green development (Chung et al., 2018; Lin and While, 2022; Zhang et al., 2022). Greenway development has become popular, bringing green imagination and new development opportunities (Pow, 2018; Pow and Neo, 2013). The development of greenways has even been upscaled into an urban model, as seen in the park city (*gongyuan chengshi*) in Chengdu. The model claims to ‘build a city inside the park’. In other words, greenways are environmental projects, while the park city is an urban development model containing the system of greenways. The model stresses the combined development of greenways, industrial parks, commercial complexes and residential communities in the whole metropolitan area. The park city is also an economic development strategy which mixes urban scenes and natural landscapes. This rationale is beyond the earlier landscaping because it combines urbanism with green space.

Following the perspective of state entrepreneurialism, this paper interrogates this park-city model. The first research question is whether the park city is for ecological preservation or land development – a key debate in China’s environmental governance (Zhang et al., 2023a). The park city creates large-scale ecological space and promotes massive urban development along greenways. The second research question regards the motivation for building the park city: whether it is for generating land profit, as seen in earlier eco-cities, or promoting a citywide economic development strategy. Similarly, ecologically oriented development has been seen in other places, such as the High Line in New York (Lang and Rothenberg, 2017). These developments are often criticised as green gentrification (Gould and Lewis, 2016; Lang and Rothenberg, 2017; Rodenbiker, 2022) and ecological modernisation (Mol et al., 2020). How is China’s eco-urbanisation similar to or different from these ecological projects?

The Chinese government has frontloaded ‘ecological civilisation’ since 2012, which aims to seek a greater harmonic relationship between humans and nature (Goron, 2018; Huang et al., 2021). Since then, a wide range of environmental projects has been developed. The park city received the endorsement from President Xi Jinping, which gives great legitimacy to the mega-urban project. All reports about the park city begin with this endorsement, dated in 2018. However, the park city is a local experiment that follows the central government’s initiative (Liu and Lo, 2023).

On the ground, the municipal government increasingly relies on land revenues to finance urban infrastructure. To generate land revenue, state-led urbanisation relocates farmers into apartment blocks in ‘concentrated villages’ (Ong, 2014; Shi and Tang, 2020). The development of ecological space thus is linked to this state-led urbanisation. The park city develops vast peri-urban areas, leading to extended urbanisation as seen in the Global South (Follmann et al., 2023; Kanai and Schindler, 2019), but still with a green twist here in China. This paper also asks to what extent this park city, or green development more generally, represents the principle of an ecological civilisation. The study explores the role of the state, its rationality and techniques in environmental governance.

This paper is organised as follows: the next section introduces the perspective of state entrepreneurialism. The third section then explains the development of the park city in Chengdu and its methodology. This paper addresses state strategic intentions in green development in the fourth section. The fifth section examines entrepreneurial development means. The sixth section discusses the limit to green state entrepreneurialism. The final section concludes the study.

## The perspective of state entrepreneurialism

State entrepreneurialism refers to a ‘series of state entrepreneurial actions to fulfil its strategic intention to maintain economic growth, stability and capital accumulation and in turn its governance capacity’ (Wu, 2023: 365). It is not a fixed model and hence can be seen as a perspective to understand the relationship between state intentionality and entrepreneurial means, including the market instrument. In an actually-existing state entrepreneurialism, this perspective includes politics of scale, regulatory flexibility and financial means (Sun et al., 2023). The distinction between state intentionality and instruments indicates the centrality of state power and the achievement of its multifaceted objectives, including economic growth, whereby state legitimacy is strengthened (Wu, 2018). This ontological understanding of Chinese urban governance stresses the overwhelming role of the state in maintaining ‘structural coherence’ – arising from the tension between capital accumulation and territorial politics (Deng, 2023; Wu, 2022; Wu and Zhang, 2022). The perspective allows us to understand why the strong role of the state co-exists in the prevalent application of market development approaches in China (Wu and Zhang, 2022). Applying this perspective to environmental governance helps to understand why authoritarian environmentalism (Beeson, 2010; Geall and Ely, 2018) operates alongside entrepreneurial governance.

It is tempting to classify the Chinese state into a dichotomy as a growth machine versus the one abiding by the statecraft of ‘ecological civilisation’. However, these two orientations towards a growth machine and authoritarian environmentalism may ‘coexist and bear different weights over time’ (Zhang et al., 2023a: 11). The contradiction between pursuing economic growth and achieving environmental objectives is only perceived rather than actually existing. By applying the perspective of state entrepreneurialism, this paper reveals the logic of environmental governance. It explains how the park-city model – built upon flexible market development – is developed to deliver the political mandate of ecological civilisation and the local government’s aspiration for economic growth.

The Chinese environmental governance is characterised by complex governmentality, not purely driven by the growth machine dynamics of capital accumulation, nor is it only political rhetoric of greenwashing (Zhang et al., 2022, 2023a). Instead, we refer to it as ‘green state entrepreneurialism’ – delivering the green objective with market operation and institutional flexibility. The perspective is thus helpful to understand the development of the park city, the evolution of development zones, new towns (Geng et al., 2023; Zhang and Gao, 2022), urban regeneration (Li, 2023; Lin et al., 2023) and the urban transformation in general in China. Further, the perspective is not a normative model to suggest the state’s success in achieving structural coherence. The tension between capital accumulation and territorial politics is perpetual. Hence, the governance model has to be constantly adjusted – the mandate of ecological civilisation arises exactly in this context, indicating the challenge rather than the accomplishment of state-centred environmental governance.

## Methodology: A case study of Chengdu’s park city model

This study chose a case study of Chengdu’s park-city model for in-depth investigation. Although the park city is now a popular planning concept in China, its experiment originated in Chengdu, which can be dated back to the designation of a peri-urban ecological zone in the early 2000s. The development has experienced three stages: greenbelt conservation, green infrastructure development through greenways and the mixed-use park city.

These stages roughly correspond to China’s changing political economy of development (Wu, 2023). In the early 2000s, China entered fast industrialisation and urbanisation as the world-factory. This stage saw the rising planning endeavour to manage the impact of industrialisation on the environment, especially urban sprawl. Starting in 2012, the second stage is a new era under President Xi Jinping’s leadership, with ‘ecological civilisation’ becoming a national policy. In this stage, industrialisation in the western region sped up since the global financial crisis in 2008. The third stage continues President Xi’s leadership, stressing ecological civilisation, environmental resilience and social governance through the People’s City (Li, 2023).

Regarding greenway development and ecological management in Chengdu, the first stage was the designation of a greenbelt along the fourth ring road of Chengdu in 2002. By then, the built-up area of Chengdu had extended beyond the third ring road and reached the fourth ring road (now the circular highway of Chengdu). Along the ring road, the peri-urban area is 198 km^2^ (hence, known as the ‘198 Area’). To prevent further urban sprawl, the master urban plan designed the peri-urban area as a greenbelt. However, land ownership was still under the control of rural communities as ‘collectively owned land’ (Kan, 2019; Smith, 2021; Tian and Guo, 2019). Rural villages engulfed by urban expansion became ‘urban villages’ (Smith, 2021; Wu et al., 2013). Rural collectively owned land has been used for non-agricultural construction. Despite persisting planning efforts, for example, designating the greenbelt as one of the ‘three important ecological areas’, over 25% of the land was developed. In short, city planning was ineffective in dealing with rampant peri-urban development (Tian and Guo, 2019).

The second stage started from the ‘peri-urban ecological zone’ legislation in 2012. The ecological zone preserves 133 km^2^ of land for ecological uses along the fourth ring road and nine radial roads. The zone forms the backbone of Jincheng Greenways in Chengdu. Unlike the concept of greenbelt to contain the metropolitan expansion, the greenways act as a system of green infrastructure, aiming to develop a series of parks. The greenway was initially based on the circular highway but was later extended into a metropolitan-wide Tianfu Greenway. The second stage aims mainly for ecological conservation and landscaping, for example the development of ‘six lakes and eight wetlands’ and an 80-km-long landscaped green corridor circulating the central city.

The third stage of the park city symbolically started in 2018 when President Xi Jinping paid an official visit to Chengdu. But, one year before his visit, the newly appointed Party Secretary of Chengdu, Fan Ruiping, relaunched the greenway project as a mega project. Under his leadership of the municipality, there were two major changes. First, in contrast to previous demolition and landscaping led by district governments, the municipal government led the new greenway project. Under the Municipal Construction Commission, a task force – the Leading Group of the Ecological Zone – has been set up to coordinate various government departments across multiple levels. The project is, in practice, implemented through a municipal development corporation, a special purpose vehicle known as the Tianfu Greenway Construction and Investment Group (hereafter, the greenway corporation). Second, beyond conservation, the new greenway project introduced mixed-use development. Rather than simply focusing on landscaping or building parks, as seen in the era of ‘six lakes and eight wetlands’ in the second stage, the approach aggressively ‘construct cities within a gigantic park’ of the whole metropolitan area. This literally introduces mixed-uses and multiple functions to the greenway. The greenway is no longer a standalone conservation project. Rather, it is associated with an extended form of urbanisation (Kanai and Schindler, 2019; Follmann et al., 2023), opening up the possibility of adjacent spaces for residential, commercial and industrial development. For example, Xinglonghu Lake was built in Tianfu New District with an investment of 37 billion RMB. Circulating the lake is the Chengdu Science City, which presents a new image of a regenerated waterfront. The ‘Unicorn Island’ is clustered with office buildings.

In 2018, the southern section of Jincheng Greenway – Jincheng Lake Park – was completed and opened to the public. The entire greenway resembles a circular-shaped park, so the ecological zone is renamed the ‘circular ecological park’. Chengdu also established a park-city management bureau to manage green spaces related to greenways, which is unusual in Chinese cities because most cities have only a forestry bureau. The effort finally received an endorsement from President Xi Jinping in 2018. The park city has become a metropolitan-wide mega-urban project. Government documents stress that Chengdu is the place of first mention (*shou ti di*) of the park-city title. The model was first experimented on and then spread to other Chinese cities. In short, the park city of Chengdu has become an ecological exemplar.

Compared with green space development in other cities in China, the park city in Chenghu has a much larger scale. It goes beyond landscaping or creating recreational paths in the Pearl River Delta (Chung et al., 2018). It is more of an urban development strategy integrating environmental objectives and green space construction. This is similar to cleaning up polluted industrial uses by township and village enterprises in Wuxi (Zhang and Wu, 2022). However, Chengdu has not encountered a significant environmental crisis for the greenway development. The development has been more growth-oriented, although its greenway development could be interpreted similarly as the ‘socio-ecological fix’ (Zhang et al., 2022).

This study draws primary data from 29 interviews conducted between 2018 and 2023. They are mainly conducted during fieldwork in 2018, a site visit and exchange visit by Chinese professionals in 2019, interviews and a focus group meeting in 2023.

## State strategic intentionality

### The central government’s mandate for ecological civilisation

The concept of ‘ecological civilisation’ was raised in the mid-2000s, emphasising a more harmonious relationship between human society and nature (Goron, 2018). Since 2012, the beginning of the era of President Xi Jinping, ecological civilisation has become a state mandate and is formally included in China’s Constitution (Pan, 2016). The new rationality aims to go beyond ‘ecological modernisation’ – achieving ecological goals through modern technologies and market-oriented development (Liu and Lo, 2023; Mol et al., 2020; Rosol et al., 2017) – by stressing the nature of civilisation with a long history. It also implies a more state-centred governance approach, with the state governing the environment through a diverse tool of governmentality. In contrast to governing the environment for economic growth and capital accumulation, the state deploys market and non-market instruments to realise its aspiration for national prosperity and civilisation. The central government’s mandate for ecological civilisation demonstrates the nature of state entrepreneurialism in environmental governance, as the state guides urban development and environmental quality with multiple extra-economic objectives (Wu et al., 2022).

Ecological civilisation, as a new rationality, tries to re-orient the local state and society away from a growth-first mentality to the balance between economic growth and ecological quality and sustainability. As a political mandate rather than a concrete policy, ecological civilisation leaves its interpretation to localities. Hence it has strengthened the central government’s role in environmental governance and created discretion in the local implementation process (Liu and Lo, 2023). Even with the recentralization of environmental governance, the central government does not exclude the objective of economic growth within the mandate of ecological civilisation. The pressure for economic growth becomes stronger locally as local governments have to balance their revenue and expenditure under a tax-sharing fiscal system.

### The local government’s entrepreneurial driver

The local governments in China are subject to a strong driver for economic growth because of a tax-sharing system, which generates pressure on local public finance. They strive to expand land revenue to fulfil development targets and local expenditures (Lin, 2014). On the other hand, local government officials pursue political careers by achieving both the economic growth target and political visibility (Guo, 2020). Earlier studies on Chinese eco-cities as new towns fully reveal this entrepreneurial characteristic of local governance (Caprotti et al., 2015; Chang et al., 2016; Chien, 2013; Liu and Lo, 2023; Xie et al., 2019).

The park-city model supports Chengdu’s ambition to become the fourth polar point of China. Although the city is associated with the history of the slow pace, rich leisure and public life around the teahouse (Stapleton, 2000; Wang, 2018), the planning target is to become a ‘new first-tier city’, joining the club of Beijing, Shanghai, Guangzhou and Shenzhen, as suggested by Wang Kai, the director of the China Academy of Urban Planning and Design (CAUPD), in an interview with a TV team from Chengdu. As a fast-growing city in West China, it has recently experienced industrialisation. Its ambition goes beyond a city in West China and is linked to the Belt and Road Initiative (BRI), another state strategy (Zhang and Wen, 2022).

The park-city model helps Chengdu realise its ambition to expand the city into a city region (Zhang et al., 2023b). The park city is a metropolitan-wide experiment with massive development of green infrastructure. According to the park-city imagination, the whole metropolitan area is perceived as a gigantic park in which multi-functional urban areas are sprinkled. Building residential estates and industrial parks in the periphery leads to profound spatial transformation. The peripheral area becomes a new geographical centre of the metropolis (Keil, 2018; Soja, 2000), as Chengdu continues to expand eastwards, aiming to develop a mega-urban region of two major cities – Chengdu and Chongqing – in Sichuan province. The park city contains a developmental meaning: ‘Once the giant park is completed, the peri-urban frontier will become the centre of Chengdu. As greenway development drives the whole city outwards, the future city will likely grow from the beltway’ (Interview, planner, 2018). Thus, the park-city model is an extended form of urbanisation (Theurillat and Graezer Bideau, 2022).

As such, the park city and its greenway component are more than a conservation project. It is a land development project in peri-urban areas, especially surrounding the Chengdu High-tech Development Zone and Tianfu New District. Additional land outside the ecological zone has been designated for development. First, although legislation sets a belt of 1,000 meters wide along the ring road under ecological protection (that is, 500 meters at each side of the motorway), in order to fund the greenway project immediately outside the designated ecological zone, 500 meters is extended at each side of the ring road as developable for the greenway company. This might generate a space of 150 km^2^ for development (an estimation confirmed by an interview, 2018). According to the plan of the peri-urban ecological zone under public consultation in 2020, the total construction land must be maintained at 53.54 km^2^. The district governments will expropriate the land of the areas. To enhance its development potential, the plot ratio of the land adjacent to the greenway is raised by 20% (Focus group meeting, November 2023). This turns out to be very critical for covering the construction cost. The construction cost will be financed by intensified land development surrounding the greenway. From the political ecology perspective, the greenway project allows the governments to gain control over the development rights in peri-urban areas.

Further, the park city also brings benefits to district governments. As mentioned, through land acquisition in earlier legislation of ecological zones, the district governments control the land in 16 small towns adjacent to the greenways. Many are small-sized rural towns. The average size is about 0.6 km^2^. They are scattered and poorly connected to the city, lacking development potential. The greenway development under the park-city model greatly enhances their accessibility and links them to the city and new district centres. The park city transforms them into new tourist places. Some begin to attract real estate projects. The greenway development corporation plans to develop 1,050 sports facilities and 600 cultural venues with the allocated land.

### Intertwined central and local strategic intentions

The park-city model combines the development intentions and strategies of the central and local governments. First, the park city originated from the national policy of urban-rural integration in China. Chengdu is a pilot city to experiment with coordinated urban-rural development in the early 2000s (Ye et al., 2013). This history helps to explain the approach of combining the park and the city. According to the park-city model, the division between rural and urban areas would disappear. The model is also said to help solve the separation between industrial and town areas (*chancheng fenli*) under the world-factory development model (Wu, 2023). According to the scenario, industrial and office workers will live in commercially built residential estates adjacent to the scenery greenway, unlike rural migrants in under-serviced urban villages in the Chinese world-factory model. In short, the rural and urban boundary disappears in this form of extended urbanisation as the park and city are blended.

Second, the park-city model is closely associated with the appointment of local government officials, subject to the party’s personnel review system. The performance evaluation drives local officials to seek the speed of development within their office tenure (Guo, 2020). They pursue land-driven capital accumulation for economic growth, which has created what is known as the ‘urban speed machine’ (Chien and Woodworth, 2018). The local leadership has driven Chengdu’s park-city development. The development evolved from earlier efforts to protect the ecology to a municipal-wide development. The development is an experiment driven by concrete motivations rather than a coherent policy of ecological civilisation. The park city was launched in 2017 by Fan Ruiping, the Party Secretary of Chengdu (March 2017 to August 2021). He came to Chengdu with some experience of Donghu Greenway project in Wuhan. Under his leadership, the greenway development evolved into a municipality-led initiative. He turned conservation into development. The park city is essentially a developmental project combined with land development along the greenway and an ambitious Tianfu New District and Eastern New Town. He also consolidated greenway development conducted by district governments into a municipal project. Using the development corporation, he managed to create a debt finance model. In this sense, the park city is not very different from earlier eco-cities because they all seek economic development. Nevertheless, the park city involves more substantial investment in green infrastructure by a new entrepreneurial approach built upon a municipal development corporation. For example, the greenway corporation invested 1005.3 million RMB in landscaping the Qinglonghu Reservoir project – part of the park city, which was completed in 2017 (Chengdu Xingcheng Investment Group Co. Ltd, 2018). Because of adopting this new development method, the park city has significantly expanded the ‘production of nature’ (Zhang et al., 2022).

Third, the park city is formulated in the context of central-local negotiation of land governance. The development is achieved through managing land development quotas (Shi and Tang, 2020). The production of green space is embedded in the political ecology of land. In China, the policy of developable land quota constrains the encroachment on rural land. The policy also prohibits the conversion of rural land into urban recreational uses. For example, a large regional greenway system is built in the Pearl River Delta, and urban parks are classified as ‘construction land use’, which is counted in the land quota. Thus the greenway project has to avoid the development of urban parks and utilise residual underdeveloped land such as riverbanks and rural lands. The paved greenways do not occupy the urban built-up area. The project inserted footpaths within existing greenbelts in rural areas and replaced existing rural roads with new greenways without requiring the quota of developable land (Chung et al., 2018). The greenway, as reflected by Ma Xiangming, the chief planner of Guangdong Planning Institute, who led the greenway plan in Guangdong, built more roads than planting trees in the first three years, and the greenway is essentially a non-motorizing system. He reveals that the initial motivation was to encourage using green space to protect it. In reality, the pace of development was too quick and more attention was paid to development than green space – a lesson to be learned.

Similarly, the policy enables the ecological zone in Chengdu to protect agricultural land. The local government demolished existing non-agricultural uses and converted them into farmland, which has become part of the preserved ecological area. The land-linkage policy created developable land quotas for the city. Counting the greenway as an ecological space helps Chengdu to save its land quotas. Chengdu learned its lesson from creating urban parks, as reflected by a planner, the ‘first project of Jincheng Lake Park was once suspended because it consumed too much construction land quota [of 400 *mu*]’, and ‘we hadn’t made use of the policy well at that time, which has been solved in this new greenway project’ (Interview, a district office, 2018). In the park-city model, these lakes are counted as reservoirs rather than urban uses. Instead of consuming the land quota, the development of greenways managed to generate additional quota by transferring the development rights outside the greenways.

In summary, compared with the green growth machines in the High Line Park in New York (Lang and Rothenberg, 2017), the park-city model is a more state-centred operation with both central and local intentionality. The park city also differs from earlier eco-cities in China. Although they both resulted in similar green gentrification and enclaved development (Caprotti et al., 2015), the dynamics in eco-cities are mainly driven by real estate capital and property speculation (Chien and Woodworth, 2022). In contrast, the emphasis on ecological values drives the park city because the state substantially invests in green infrastructure and strives to capitalise on the ecological value. Nevertheless, given a significant investment in green infrastructure, it is increasingly imperative for the municipal government to generate revenue through green-led economic development. The funding pressure requires the local state to mobilise capital and capitalise on land value by creating ‘urban scenes’. As a result, the green infrastructure is centred upon the state development strategy.

## Entrepreneurial development means

### Land expropriation through transferable property rights

Large-scale demolition of rural settlements was initiated to build the greenway and ecological zone (Du, 2022; Smith, 2021). At the time of legislation in 2012, about 240,000 farmers still lived within the ‘198 peri-urban area’. Under the legislation, the municipal government set up boundary marks. The district governments were responsible for implementing land expropriation. The task was assisted by grassroots-level governments – the street offices. From 2012 to 2017, district governments demolished 410.9 million m^2^ of buildings in urban villages and reduced the total area of rural construction land to 20 km^2^. In 2017, 1.7 million m^2^ of rural housing was demolished and 1.87 million m^2^ of resettlement housing was built (Zeng, 2017). Concentrating rural settlements into new resettlement housing of new multi-floor buildings has been widely seen in China (Ong, 2014). Ecological conservation is used to justify relocation and resettlement (Rodenbiker, 2020).

According to the conservation policy, construction land under rural collective ownership was expropriated at 2 to 1 in the area from 500 m to the ring road, which is the ‘priority zone of ecological protection’. Rural communities can get half the size of the original demolished land at a new place. Compensation was paid more than the compulsory purchase of agricultural land for infrastructure construction to make the project easier to implement. District governments covered the cost of compensation and rehousing farmers. In return, district governments managed to gain control over land ownership. The land is restricted within the zone, but the land quotes have been generated. The greenway is expected to significantly raise the value of the land along the greenway.

The ecological zone project managed to build 27 km^2^ of forests and 4.1 km^2^ of wetlands. In addition, it absorbed about 100 km^2^ of agricultural land, of which 35.2 km^2^ is designated as ‘basic agricultural land’ under strict protection of central government policy. However, some agricultural land has been used for ‘agricultural landscape’, such as fruit, tea, flowers and oilseeds. This is particularly problematic as some ‘basic agricultural land’ has been converted for non-agricultural use. According to our estimation, the agricultural land plus newly built forest and wetland added up to roughly 133 km^2^ under conservation.

The development of ecological zones also protects the land from being constructed on. It demolished rural construction land into broadly ecological land. In this case, agricultural land is treated as part of the ecological system. The then director of Chengdu Planning Bureau, Ms Zeng Jiuli, used the word ‘restoration’ to describe the creation of basic agricultural land. The outcome of restoration increased the size of agricultural land using this name. According to the state policy of ‘linked land’ (i.e., transfer of development rights) (Du, 2022; Ong, 2014; Shi and Tang, 2020), an equivalent size of developable land quota might be generated for urban development outside the greenway. They may be in adjacent concentrated areas.

However, driven by the land policy, restoration does not produce agricultural land for crop production. The land was used for landscaping. Including basic agricultural land in the ecological zone creates a potential problem as the land was, in practice, used for landscaping in the ecological zone rather than agricultural production. The municipal government deliberately confuses ecological land and agricultural land to realise its intention to create an ecological landscape rather than the agricultural field. This contravenes the strict rule of agricultural land protection. The misuse of land received the forceful intervention of the central government. In 2023, the Ministry of Natural Resources required Chengdu to remove landscaping grassland to restore agricultural land.

Reassigning the development of greenways as a municipality-led mega project has some challenges because land resources created in the previous stage are not in the hands of the municipal government or its agency. Inside the ecological zone, the developer, the greenway development corporation, joined the project in 2017 and controlled only a very small amount of land. To solve the financial constraint, district governments are required to contribute to greenway construction. They must pay the greenway company a fixed rate of 1 million RMB per *mu* from the proceeds of the land sold.

According to our informant, the land in the hands of the greenway corporation only amounted to 1.17 km^2^ in 2020. The land is mainly for facilities and auxiliary buildings. In a planning forum (The Annual Meeting of the Urban Planning Society of China in 2020), the director of the greenway corporation confirmed that 1.5% of land in the greenway is for construction purposes, among which 30% is for public services and 70% is commercial. However, these land plots are small and ‘dispersed in the zone like sesames’, which can hardly generate a profit (interview, a business manager, 2018).

Under ecological conservation, the government managed to acquire the land, and through planning, the development right has been transferred from rural communities to the municipal government. The majority of land assets are in the hands of multiple actors, including district governments, and a small proportion is allocated to municipal-level state-owned enterprises. This means the greenway corporation alone cannot capture land value. It acts for the municipal government to develop the greenway. Rural communities also received some developable land, but the overall quantity of land has been reduced by half. The density of resettlements is higher than that of original rural settlements, which means the compensated floor space is achieved through higher densities.

In short, under ecological legislation, the district governments managed to expropriate the land development rights within the ecological zone. The conservation project before the park city had been slow and halted around 2016 because of the lack of investment. The development of the greenway thus relies on a new finance model.

### Financing green infrastructure by financial means

The mega green infrastructure project is estimated to require a massive investment of 47 billion RMB (see later figures ranging from 41 to 47 billion RMB). By comparison, Chengdu’s fiscal revenue is 126.78 billion RMB plus 134.02 billion RMB fiscal transfer from the central government. Its GDP is 2,082 billion RMB, according to the Finance Bureau of Chengdu. How can it afford such a large investment? This is in contrast to a more decentralised model of governance of greenway development in the Pearl River Delta (PRD) (Chung et al., 2018). Chengdu adopts an integrated and coordinated approach by establishing a municipal development corporation for green infrastructure investment and construction. In the PRD greenway, each district or county government is responsible for mobilising investment and building the section of greenways in its jurisdiction. In Chengdu, the construction of the Tianfu Greenway project is a mega-urban project under the municipal government. The municipal government plays a strong leadership role and has established the greenway corporation to implement the project.

To implement the mega project, Chengdu Tianfu Greenway Construction Investment Group was established as a wholly-owned subsidiary of Chengdu Xingcheng Investment Group – a municipal investment platform. The latter is a state-owned enterprise under the Chengdu State Administration of State-owned Assets Commission (SASAC) (Chengdu Xingcheng Investment Group Co. Ltd, 2018). In China, the development corporation is a state-owned enterprise and the state’s agent operating in the market (Feng et al., 2022). The group plays a dual role as a state-owned development company and a local government investment vehicle (known as *chengtou*). The company only builds the greenway for the municipality as an agent of construction (in Chinese terms, the corporation built the greenway for the government, *dai jian*). It should receive reimbursement for its investment. This is because after the central government tightened local finance platforms in 2016, local government financial vehicles cannot act for the government to raise the debt (Li et al., 2023). Chengdu Xingcheng Investment Group is essentially a *chengtou*, bearing a corporation status. Its debt is thus not treated as government debt but rather as a corporate debt.

As a subsidiary of Xingcheng Investment Group, the greenway corporation managed to access the capital market for greenway construction, even though the greenway project could not generate immediate cash flow. In other words, investment in the greenway project is not evaluated through its own profitability or the credibility of the corporation. Instead, the greenway corporation managed to access finance through the overall portfolio of the investment platform. Under Xingcheng, property development companies such as Xincheng Renju managed to obtain substantial land along the ring road. In essence, this allows cross-subsidy of greenway construction from property development and business of utility companies. Financial borrowing is channelled through Xingcheng Investment Group.

Land value capture is expected to finance the greenway project. Development projects in the prioritized conservation zone along the greenway are required to contribute one million RMB per *mu* to the project (interview, 2018; 1 *mu* = 15 hectares). In Chengdu, the income from land sales is divided between the district and municipal government according to the proportions of 20% to 80%, indicating a high concentration of captured land value in the hands of the municipal government. The municipal government receives the larger amount. The greenway construction led to an increase in land value and, hence, increased land incomes. In 2016, a plot of land in the area was sold at 20 million RMB per *mu* during the real estate boom, breaking the record for land sale in Chengdu. The land sale was expected to generate a land revenue of 37 billion RMB. The capital was planned to fund the construction of the entire greenway project. Another source suggests that in total 40 billion RMB will be invested when the project is completed in 2027. Up to now, 10 billion RMB has already been invested (Interview, planner, November 2023). The official figure for the construction cost of the greenway is 41.5 billion RMB (Chengdu Xingcheng Investment Group Co. Ltd, 2018), which has been confirmed again by the greenway director in a public planning forum.

Despite massive investment, the greenway development corporation has limited land assets. Its balance sheet includes the management of facilities. It does not possess the land along the greenway and cannot perform land value capture itself. According to the director of the greenway development corporation in a public planning forum held in 2020, although the corporation produces an annual business income of 2.8 billion RMB through land development in the greenway, its annual profit is only 0.1 billion RMB. Such a level of profitability would not allow large-scale investments to be performed without financial injection from the government or other sources. In other words, the capture of land value is carried out at the greenway development corporation level. It has to receive government funding for greenway construction.

Chengdu adopted a ‘double-circulation’ strategy for financing the greenway. The first circulation covers the maintenance cost of greenways by bestowing a limited amount of land to the greenway development corporation to build commercial and operational facilities. For this purpose, 1.5 km^2^ of land is relocated to the greenway corporation for small development inside the greenway, supposedly for park maintenance. According to the adjustment made in 2020 for the ecological zone, the revised plan allocated the development quota to the greenway corporation while keeping the overall land of 133.11 km^2^ as ecological land.

The second circulation uses mixed land development to generate land income to cover investment costs. It is confirmed that the construction land of rural collectives was reduced to 20 km^2^ (Zeng, 2017), which means at least 23.54 km^2^ of construction land is now under the control of governments for development. According to the project finance forecast, the government hope to sell the land in these mixed land developments to cover the development cost.

This debt-based model of green infrastructure financing generates significant financial pressure on local government public finance (Li et al., 2023; Tsui, 2011). Assuming an interest rate of 3%, the annual interest for the development cost of 41.5 billion RMB is 1.245 billion RMB, although the cost is unlikely to be incurred simultaneously and is recovered on a rolling basis. The greenway project would only be successful if it could generate a significant tax increase for the government. The current integrated development approach depends on increased government revenue through mixed-use development.

### Creating ‘urban scenes’ for consumption and ecologically oriented development

Branded with ecological civilisation, the park city aims to present a new urban development model beyond China’s world-factory. For example, this model has been extensively criticised in eco-city development (Chien, 2013). Its ecological value is dubious and often criticised as greenwashing. In Chengdu, there is an effort to green production (Zhang et al., 2022). Green production is associated with mixed-use development.

The development strategy combines consumption-driven green development with financialisation (Theurillat and Graezer Bideau, 2022). According to the chairperson of the greenway corporation, Kang Ying, the park city is not a ‘text’ but a ‘scene’ of urban life, which Chengdu citizens can enter. It is a scene that can be appreciatively looked at, can be participated in and can be consumed. These urban scenes include sports, museums, panda parks and traditional West Sichuan forest-residential compounds (*linpan*). When asked why the concept of a park city emerged in Chengdu, Wang Kai, the director of CAUPD, explained that Chengdu is located in superior geographical areas where traditionally forests and residential compounds were combined into these forest-residential compounds. The park city begins with an ecology that takes on developmental meaning and hopes to restore the relationship between the urban and natural environment (Rodenbiker, 2020). Reflecting on the ecology, the park city conserves the natural environment and adds new green spaces, modifies existing green areas through landscaping and opens them up for consumption. Though a grand project, the park city intends to create more than a green spectacular, which strives to combine ecology and everyday urban life.

As Zeng Jiuli, the former director of the City Planning Bureau of Chengdu, and her colleagues explain, this model is different from an industrial city in which the logic was

to attract enterprises through the land, then build residential areas, then subsidise public facilities construction with the modest profit from selling the land. Now, in the new park city, the logic follows the order from constructing the [urban] scene, attracting people, managing the city, to promoting business, which means the development of parks, greenways, public services facilities, and key functional infrastructure first, and then attract talent agglomeration, rapidly raising the overall value of the area, leading to adjacent commercial, industrial and residential development, to maximise the overall benefit, and finally to realise ‘the park first, then the city; urban living first, and then production’ (Zeng et al., 2021).

According to this model, the park city represents a more ‘advanced’ urbanisation model. This development strategy’s crucial component is constructing urban ‘scenes’ (*chang jing*), which can be understood as a venue with good landscape and interesting activities. As a landscape designer reflects, the task deliberately aims to create ‘focal points’, ‘scenes’ and ‘venues’ for visitors to stay (Interview, 2018). The carefully prepared activities in these venues turn them into consumers. This is apparently associated with tourism, other consumption and even production themes, such as commercial complex and intelligent production. In short, this is a scene of urbanisation combining ecological spaces and vibrant urbanism.

A complete list of intended corporation projects issued by the greenway corporation in 2020 covers almost every aspect of daily life, from supermarkets, souvenir stores, restaurants and fast food, hotels, leisure agriculture (e.g., happy farms), recreation and bookstores. Associated with the greenway development, cycling businesses such as bike sales, repair and storage have become a new industry. For sports activities in Chengdu, the value of the sports industry exceeded 80 billion RMB, and the average per capita spending is 2,298 RMB, accounting for 8.7% of per capita expenditure. Beyond everyday consumption, tourism and leisure, and spectacular sports, the new ecological zone plan under public consultation in 2020 depicts a full picture of these scenes.

The park-city strategy hopes the urban scene would create urbanism in these edge cities in peripheral Chengdu. The development accelerated the pace of suburbanisation. The construction of Chengdu High-tech Zone and Tianfu New District, a national-level development zone, proceeded on an unprecedented scale. The development of new areas has created a polycentric urban structure. As explained by Wang Xiaoqi, the current director of the Chengdu Planning Institute, Chengdu has experienced the development from ecological conservation to ecological parks, from pushing development away from ecological space to embracing the park (presentation in the Public Forum of Urban Planning Society of China, 2020). The task is thus to realise the ecological value through ‘Park +’, which means extending the park into a wider scope of urban activities. He stresses the need to blur the distinction between the park and city (in this sense, it is a park city) and advocates a blended approach called eco-urbanisation. He proposes ‘ecologically oriented development’ (EOD) and transit-oriented development (TOD) to optimise the metropolitan spatial structure. These green-infrastructure-driven and transit-oriented developments have been widely seen in many Chinese cities (Shen and Wu, 2017; Zhang and Wu, 2022). Based on the ecological space, nine industrial clusters have been planned or created through ‘eco+industries’. Further, 11 residential areas are constructed with the concept of ‘eco+living’. Some are located near lakes and parks. The linear greenways and high-speed roads connect these clusters as the non-motorized system. Moreover, Chengdu attempts to embrace the new economy by constructing a ‘Unicorn Island’, now renamed the high-tech eco-island and masterly designed by Zaha Hadid Architects, around the man-made Xinglonghu Lake, where President Xi Jinping endorsed the concept of the park city.

In short, flexible market development approaches include land consolidation and converting land into government control, creating multiple urban functions and mixed uses, taking the extended form of urbanisation, especially blending real estate, industry and nature at a massive scale beyond the singular city. The park-city model is known as an overall ‘ecologically oriented development’.

## The challenge of green state entrepreneurialism in the post-pandemic era

The ecological-oriented approach is largely a development approach. The government sells the land near the greenway and ecological spaces to raise the capital to finance green infrastructure. Although the discourse of attracting people presents a spillover effect of greenways, the impact on land value for the government to capture increased land value or the industries, which are supposed to raise the fiscal income, is less certain. Land income is the main source for investors in these portfolios to generate the project return. Invented in Chengdu, the concept of the park city is allegedly mimicked by other Chinese cities. For example, Shanghai is said to be learning Chengdu to become a park city by 2035. However, by measuring GDP, R&D input and per capita household income, Chengdu still lags behind the first-tier cities.

The greenway development corporation does not generate the profit volume to recover the primary infrastructure construction cost. At most, it hopes to rely on the limited land assets inside the greenway to cover its operation cost. As mentioned, it acts on an ‘agency basis’, which means the government pays for its services. This implies that the greenway needs to be financed through public finance under taxation. However, the financial model is not based on fiscal revenue, as mentioned earlier. It relies on the land finance model. The government hopes to sell the land to cover the construction of green infrastructure. The financial model is justified by the increase in the land value from which investment can be recovered. However, this becomes very uncertain under the post-pandemic macroeconomic conditions and real estate downturn.

When the greenway lacks financial viability, the project has to be justified by its contribution to future taxation increases through an economic development strategy. This strategy stresses the role of green infrastructure in the economic development of Chengdu, suggesting greenways contribute to a good living environment and thus attract a talented workforce, which in turn creates demand for properties and attracts industries and businesses in the sequence of people, city and industry (*ren*, *cheng*, *chan*). The sequence of development hopes to increase the tax income eventually. The discourse justifies the investment in ecological space due to its economic value. However, the causal relationship between people, the city and industries is only perceived rather than actually existing. Its realisation is full of uncertainty.

Because of this financial imperative, although the greenway focuses on ecological value, it is a development project. The green development is a tool for land-driven capital accumulation. The project is similar to ‘property-led regeneration’ such as the waterfront development of Canary Wharf in London, which has been widely practiced in Western economies in the 1990s (Fainstein, 2001), albeit with a green component. Since then, new financing methods, such as tax increment financing (TIF), have been used in urban development in Western economies (Weber, 2010). In contrast, the greenway project is only a development strategy, assuming investment in green infrastructure would enhance the quality of the residential environment, attracting a talented workforce to boost its new economies. This green development strategy expects future tax increases. However, the government revenue prospect is uncertain. Essentially, it speculates on the tax increase to justify the massive investment in green infrastructure. The Chinese green state entrepreneurialism thus remains a financialised governance. Rather than a calculative figure, the tax increase is only a discourse to achieve a ‘jump-start development’ (*kuayueshi fazhan*), which has become increasingly risky in the post-pandemic period.

## Discussion

This paper offers a more nuanced understanding of green-driven development in China, which goes beyond its past practices of greenwashing – profit-making in real estate development and green boasting without creating green spaces. Here, the new approach reveals the state mobilising substantial investment in green spaces to capture the economic value of ecological spaces. The ecological zone and later park development in Chengdu are integral aspects of this green state entrepreneurialism. The park city is more than greenwashing or a green imaginary. The proposed green solution involves the material construction of green infrastructures – greenways, parks, wetlands and lakes – which is adopted not simply for the sake of its ecological value but mainly because of its catalytic effect on urban development.

The greenway creates an exclusive zone for ecological uses. However, unlike a greenbelt, it does not aim to contain urban expansion. Instead, ‘like a magnet’, it serves an opposite function: to draw people into peri-urban and even exurban areas (interview, a landscape architect, 2018). Since greenway development, Chengdu has further experienced rapid urban expansion. The city has leaped over the circular ecological zone into the Tianfu New District. It even jumps over the Longquan mountain and develops a new urban district – Eastern New Town in the former city of Jianyang, near Chengdu’s second airport – the Tianfu International Airport.

The official presentation of the park city constantly stresses that it is an exemplar of ‘ecological civilisation’ envisioned by the central government (Chung and Xu, 2021; Geall and Ely, 2018; Huang et al., 2021; Kostka and Zhang, 2018; Liu and Lo, 2023). It is allegedly aligned with the central government policy. Reflecting on the question: can the park city of Chengdu represent ecological civilisation? This paper reveals its material motivations. The common explanation is that the local government pursues land revenue (Lin, 2014; Tao et al., 2010). It seems to be a straightforward explanation. Nevertheless, although the park city requires mobilising development finance from land, land profit is not a direct cause. There are easier ways to generate land values than the costly greenway project. Massive investment in green infrastructure generates enormous financial pressure on the local government. Similarly, the state has multiple objectives with the financialisation approach to affordable housing development in Shanghai (Shen et al., 2022). Neither is investing in green infrastructure in Chengdu a purely political motivation to implement the ecological civilisation of the central government.

Beyond either a land profit motivation or ecological mission, this article reveals, through grounded field observation, that it is a strategy of green state entrepreneurialism. State entrepreneurialism is to achieve the state’s multiple objectives through entrepreneurial statecraft and market development means (Wu and Zhang, 2022). The green here is both the objective (fulfilling a state mandate) and means (as green infrastructure). The explanation may be much simpler: to develop a grand project to demonstrate the state’s achievement – to fulfil ecological civilisation and economic development at the same time. Through a financial approach, the project promotes local economic development and fulfils its ecological mandate without requiring direct government finance. However, implementing the park city reveals a process of using ecological spaces to promote local economic development.

The strategy of green state entrepreneurialism is not an entirely top-down one. It delivers an explicit local economic development strategy. The development strategy has been shifted from ‘attracting investment’ to ‘attracting people’ in the order of ‘people-city-industry’. The strategy does not explicitly connect to ecology. But, it justifies the production of nature. Ecological improvement is regarded as indispensable to implementing the economic development strategy. The strategy implies that the ecologically superior environment attracts talents in high-value-added sectors. In peripheral areas where rural ecosystems are the dominant landscape, ecological conservation and upgrading become critical steps toward building a high-quality urban environment. This is similar to ecological modernisation (Liu and Lo, 2023; Mol et al., 2020; Xie et al., 2019) without assuming the central role of the private sector.

The peri-urban areas were quite chaotic in land uses and detrimental to the environment due to urban expansion in the post-reform period. They have been transformed into aesthetically pleasing new districts. Although the land is under ecological conservation, the development of the park city has effectively turned nature (ecology) into a man-made green environment. As such, green state entrepreneurialism also sees a process of ‘green gentrification’ (Du, 2022; Gould and Lewis, 2016; Rodenbiker, 2022). Although rural farmers remain in the peri-urban area, their habitat is replaced with landscaped parks and middle-class residential areas mixed with office development (Zhang and Wu, 2022).

As stressed in many government documents and presentations, the park city does not just build parks but promotes comprehensive mixed-use development. At the micro-scale of the greenway itself, it combines recreation, physical exercise, sports and leisure scenes. The mesoscale of clustered development areas combines green space, residential development, commercial and office complexes, and technological industries. At the macroscale of the whole metropolitan region, the municipality extends to connect with a nearby Jianyang county, which was annexed by Chengdu in 2016. The peri-urban development, along with green space creation, is part of an overall expansion of the metropolitan area of Chengdu from five districts to eleven districts. The greenway no longer serves as a greenbelt to contain the spatial expansion of Chengdu. It is a ‘combinational urbanisation’ process that facilitates city-region development, in which Chengdu is an ecological park.

Under green state entrepreneurialism, development is achieved through land financing. It removes informal village development in the peri-urban area under ecological conservation, strengthening the state’s power (Rodenbiker, 2020). Under the ‘land linkage’ policy – a Chinese version of the transfer of development rights (TDR) – ecological protection generates the quota of land development for adjacent areas (Shi and Tang, 2020). This transfer of land quota is accompanied by the change of development actors from rural communities to district and municipal governments and then to the municipally owned development corporation. The outcome effectively consolidates the development right in the hands of the government and its agencies in the peri-urban areas. On the other hand, the implementation also takes a ‘governance’ approach. In contrast to the earlier phase of ecological zoning led by district governments, the municipal government does not directly participate in its construction but coordinates its governance through a market agent – the development corporation. The development combines state intentions and market operations.

The most striking feature of green state entrepreneurialism is that development and conservation are no longer regarded as contradictory, as is often perceived in city planning principles. In studying China’s environmental governance, a ‘local implementation deficit’ has been noted for a long time (Kostka and Nahm, 2017), showing that the local government traded off between economic development and pollution control (Wang et al., 2023). Now, this green-driven development stresses the aesthetic landscape of ecological space (Lang and Rothenberg, 2017). The park city introduces the ‘scenes’ of urbanism in which people can stay and participate in activities such as sports and leisure. These scenes of urban life accompany population densification in the more mature suburbs and new towns. The park city is more than a collection of parks; it is also an interconnected urban area (Keil, 2018). It is a multi-functional city that combines culture, sports, tourism, commerce and new technologies. The combination of ‘ecological space’ and ‘urban scenes’ is imperative and synergistic with each other. The park-city model is thus a local reinterpretation of the recent central government political ethos of ‘ecological civilisation’ and ‘cities for the people’ (Li, 2023; Li and Zhong, 2021; Wu et al., 2022) through concrete green and urban projects. While ecological civilisation is not a direct cause of the mega-ecological project, the ethos lends some legitimacy to a local initiative (Chung and Xu, 2021; Wu et al., 2022; Zhang et al., 2022).

There are similarities between the park city and neoliberal green urbanism, such as the High Line in New York, which is, as Lang and Rothenberg (2017: 1743) observed, ‘ecologically inspired and aesthetically designed leisure, consumption and tourist spaces based upon the principles of Landscape Urbanism and ideas about sustainable park design’. However, the park-city model occurs at a much larger scale in the whole metropolitan area. The upscaled greenway system is a massive investment in green infrastructure. The role of the state is more visible. The park-city strategy adopted since 2018 continues this trend of investing in green infrastructure and land value capture. Its focus has shifted from green investment to mixed-use development to capitalise on the improved environmental quality. From greenways to the park city, their implementation depends on future land value capture, which remains uncertain in post-pandemic China. Because of this uncertainty, this aggressive green infrastructure development is increasingly difficult to implement and brings financial risks. These challenges expose the limit to green state entrepreneurialism.

## Conclusion

The perspective of state entrepreneurialism highlights the role of the state in governance, characterised by state centrality (Wu and Zhang, 2022). Embodied in environmental governance, green state entrepreneurialism means that the state resorts to various market approaches and institutional flexibility to achieve state rationalities, including economic developmentalism and green environmentalism. These objectives are not necessarily mutually exclusive, as seen in the park-city model. In other words, Chengdu’s park-city model does contain an extra-economic element, more than profiting from land (Zhang et al., 2022). But in essence it is also a new growth machine (Li, 2023). This is because the green development has to be financed by industrial, residential and commercial development along the greenway and in an extended park city. For the municipal government, especially for Tianfu New District, the intention to introduce green space for economic development is even more significant. Therefore, the park-city model is not a de-growth strategy.

The outcome is a strengthened state power in urban governance, as evident in the residential relocation of local farmers for making green spaces, designating agricultural land into landscapes and the rise of municipal development corporations in financing and constructing greenways. The park city expands the quantity of landscaped green areas along with urban expansion. Like all mega-urban projects, the development significantly impacts the life of original farmers living in the fringe area (Wang and Wu, 2019). The park city has also created new spaces for real estate developers for property development and for the district governments and national new areas to develop eco-industrial parks. The ‘urban scene’ of the park city promotes tourism, attracts visitors and injects urbanism into suburban areas for the middle-class residents. While these dynamics seem to synergise green urbanism and economic development, tensions are inherent in the park-city model.

Building the park city is costly and may not generate the expected causal effect ‘from attracting people, developing properties, to expanding industries’. This creates a debt-financing approach – financialised state entrepreneurialism – creating greater financial risks. The policy implication is that this model might be difficult to replicate in other places, especially when the real estate market faces a downward trajectory. The park-city model of Chengdu has been produced at a specific historical conjuncture – the strengthened ethos of ‘ecological civilisation’ and geographical specificity – Chengdu is an emerging economic centre in China’s western region. Using green space development to promote emerging industries is not a proven formula. Implementing this at a larger regional scale is even more challenging.

The theoretical implications of the park-city model for state entrepreneurialism are twofold. First, the park-city model shows multiple objectives in China’s environmental governance. There has been debate about whether China’s environmental governance can be seen as a model of neoliberalism – the growth-first through greenwashing. However, the park-city model reveals extra-economic motivations. It is presented as a combination of ecological objectives with economic development, or at least these objectives are not confrontational in the park+ discourse. The environment improvement is a ‘sustainability fix’ (Zhang and Wu, 2022; Zhang et al., 2022), contributing to rather than prohibiting economic development.

Second, the park-city model shows the limit to green state entrepreneurialism, as there is no way to guarantee its success, especially during the economic downturn. With multiple objectives, as shown in green state entrepreneurialism – combining green urbanism and entrepreneurial development approaches – the ‘planning centrality, market instruments’ model does not solve the tension between these two. This is a deeper reason why state intentions might not be actually fulfilled. The logic presented through state entrepreneurialism as the analytical perspective shows an internal tension between multiple objectives. Implementing green urbanism through the park-city model encounters different interpretations and tactics. Chengdu may face different objectives, such as protecting agricultural land and the requirement for greater environmental protection under ecological civilisation and adapting them with varying weights at different times. As the park-city model reveals, there are different governmentalities: greenwashing might be one at a time, and green place branding might be another at a different historical conjuncture. Chengdu downweighed, tactically, the protection of agricultural land for the park city. State entrepreneurialism as a governance model is limited by its instrumental treatment of market mechanisms. But, when a financialised approach is used, especially for delivering the environmental outcome without a direct financial return, the logic of capital accumulation still exists. The park-city model of Chengdu also reveals the value of state entrepreneurialism as a perspective because it stresses both the state intentionality and entrepreneurialism related to market operation and institutional flexibility. The perspective explains the deep contradiction in China’s urban governance by stressing both elements and their dialectical relationship.
